# Proceedings of the Richmond (Va.) Academy of Medicine and Surgery—A Case of Laparotomy—Osteo-Sarcoma of Femur, Amputation—Triplets

**Published:** 1891-01

**Authors:** Jas N. Ellis

**Affiliations:** Reporter


### PROCEEDINGS OF THE ACADEMY OF MEDICINE AND SURGERY.

RICHMOND, VA., NOV. 25, 1890.

President W. W. Parker M. D., in the chair. The following surgical operations were reported by Dr. George E. Meredeth : a diagnosis of ovarian tumor was made in the case of Miss C. age 29, and on the eighth of October last assisted by Drs. Baker and Jas. N. Ellis., the usual incision through the linsa alba was made down to the peritoneum, after all hemorrhage was arrested the peritoneal cavity was opened, the hand being then introduced, both ovarian regions were explored, revealing the presence of a tumor of the left ovary containing about 2 ounces of fluid. A silk ligature was passed, and the tumor and ovary removed. The edges of the peritoneum were then approximated by fine cat-gut sutures, and the external incision closed with silk. Antiseptic dressings were then applied, and patient put to bed.

She was given tonics and liquid diet, with morphine, for several days, temperature rose to 102 2-5, made a rapid recovery. All of her previous bad health disappeared, and she is now walking about for the first time in years.

2nd case. White man age 35 osteo-sarcoma of the femur, amputation of, at the hip, after the method suggested by Ferneau Jordan, the dressings being removed, on the sixth day, one month subsequently the patient was well and riding about.

3d case was that of a white woman age 29, twenty years previously had been salivated, followed by ulceration and the formation of dense cicatricial bands, and closure of the jaws, through an opening made by extracting teeth she took liquid and soft food. At the time of first visit, was suffering from tooth ache, swelling was great, and suppuration seemed imminent, the following operation was decided upon: an incision was made, commencing midway between the anterial border of the sterno-cleido-mastoid, and the angle of jaw, and carried forward just beneath and parallel to the lower border of the symphisis, when it was extended vertically upwards to within 1-2 an inch of the red line of the lip. This flap so to speak, was dissected up, preserving the periosteal attachments of the muscles; and the corresponding surface of the inferior maxilla laid bare. The cicatricial bands on the right side were then divided. The teeth on this side were then removed, and passing two fingers as guide, through the opening thus made ,the bands on the opposite side were severed. The jaws were now forced apart and the teeth on the right side drawn, which were all decayed and abscessed, necrosis of the left side of the inferior maxilla. After removing the dead bone, the wounds were stitched together and dressings applied. The mouth was syringed twice daily with a one-two-thousandth solution of bichloride. The wound healed readily, and the patient now has almost perfect use of his jaws, and contemplates artificial teeth.

Dr. W. W. Parker had seen a similar case, in which cicatricial bands formed as a consequence of salivation. Mr. Blair recalled a case of complete anchylosis of the tempero-maxillary articulation, resulting from the injury received in a fall. When first seen by Dr. Hunter McGuire fatty degeneration of the muscles of mastication had occurred, and he removed a section of the inferior maxilla, making a V shaped opening for the introduction of food.

TRIPLETS.

Dr. J. W. Henson had recently been called to see a woman with a very much distended abdomen, oedema of lower extremities and of the tissue above the pubes. He diagnosed pregnancy with hydro-amnii. Two weeks subsequent she was delivered of male triplets—the smallest coming first, the second size next, and the largest last. Vertex, foot and breech was the order in which they presented. There were two placentas, the first and second child having one in common. They all died immediately.

Dr. Charles W. Shields examined a man at his office this evening whose case presented some peculiar features. The patient was above middle age, and the subject of aural catarrh with impaired hearing.

For certain sounds, his sense of hearing is almost normal, but for others it is practically nothing. Upon one occasion he was in the room with a canary and a clock. The bird was a fine songster and his shrill notes so annoyed others present that they would place their hands to their ears to drown the sound. But while the doctor's patient could distinctly hear the ticking of the clock he could not hear the bird at all. The theory casually submitted as explaining this deficiency is that there is destruction of the more sensitive portion of the labyrinth.

The doctor next spoke of an operation for the removal of superabundant tissue above the upper eyelids which he has recently performed.

He removed an oval piece of skin one by one and one half inch in size, from both lids, and brought the margins together with stitches, and expects a good result.

Dr. Wm. S. Gordon is informed that certain deaf people hear best on R. R. trains, and wishes Dr. Shields to give the true explanation of this. He has been impressed by the fact that certain tones of the voice are more readily appreciated by those whose sense of hearing is impaired.

Dr. Shields said that the majority of deaf people hear best that which is uttered in a low, distinct voice, loud tones causing a confusion of sounds upon the drum membrane. Thinks the reason some people hear best on a railroad train is that when in the midst of other noises the auditory apparatus is stimulated to a degree of tension which keeps the drum munbrane in a better receptive condition. This is generally supposed by deaf people to be incouraging, but the doctor considers it an unfavorable symptom.

Dr. W. W. Parker was called, about the middle of the month, to see a boy age 6, with a bad case of diptheria, he died of exhaustion the third day of the attack. The premises were thoroughly disinfected. But one week subsequently a girl age 8, developed a violent case, which also ended in death. The constitutional symptoms in this case were particularly severe, and the local swelling extended entirely across the larynx as if a cord had been drawn around the throat.

The usual treatment was followed with active stimulation. A severe hemorrhage from the nose was easily arrested by Parker's Plug, a method first suggested and practiced by the speaker. On visiting the patient yesterday, the swelling had subsided, appetite better, diphtheretic membrane nearly gone, mucous membrane looking healthy, secretions from nasal mucous membrane normal, pulse better. To-day however, the doctor was surprised to learn that the patient had suddenly died. The termination of this case impresses upon him the necessity for a very guarded prognoses in bad cases of diptheria. He has attended six cases of diptheria recently, with mortality of five out of the six. Thinks this epidemic of a very malignant type.

Dr. Shields has seen more cases of paralysis of the muscles of tonsils and throat following diptheria in this epidemic than ever before, coming on immediately when the patient is not then convalescent. In one case the paralysis of the ocular muscles occurred three weeks after recovery, and was followed still later by paralysis of the limbs. When the muscles of deglutition are affected, the food has to be given through a tube which is passed below the larynx. He gives sulphate strychnia in 1-20 grain doses until the physiological effect is produced, using, at the same time, the faradic current along the pharyngeal muscles. It usually takes several weeks, and sometimes months, to effect a cure.

The doctor next mentioned the case of a man age 63, who first experienced difficulty in speaking four or five months ago. This was soon followed by difficulty in swallowing. He put himself in charge of a Washington specialist who had the reputation of being a very careful and skilful man. He discovering ulceration of the glotis, and vocal cords diagnosed carcinoma and predicated a rapidly fatal termination The patient subsequently came to the speaker and was taken to the Retreat for the Sick ; one half of the epiglottis was eaten away, and one of the vocal cords gone. Doubted Carcinoma, especially as he had no pain, and suspecting tuberculoses examined the chest, finding one of the lungs consolidated, was kept alive for some time by feeding him through a tube and ultimately died from accumulation of mucous, and consequently suffocated. This case is interesting, because the tubercular trouble primarily manifested itself in the larnyx, which is exceptional.

JAS N. ELLIS, M. D., Reporter.

---

### THE MATTISON PRIZE.

### OPIUM ADDICTION AS RELATED TO RENAL DISEASE.

### A PRIZE OF FOUR HUNDRED DOLLARS.

With the object of advancing scientffic study and settling a now mooted question, Dr. J. B. Mattison, of Brooklyn, offers a prize of $400 for the best paper on "Opium Addiction as Related to Renal Disease," based upon these queries:

Will the habitual use of opium, in any form, produce organic renal disease?

If so, what lesion is most likely?

What is the rationale?

The contest is to be open for two years from Dec. 1, 1890, to either sex, and any school or language.

The prize paper is to belong to the American Association for the Cure of Inebriety, and be published in a New York medical journal, BROOKLYN MEDICAL JOURNAL, and *Journal of Inebriety.*

Other papers presented are to be published in some leading medical journal, as their authors may select,

All Papers are to be in possession of the Chairman of Award Committee, on, or before January 1, 1893.

The Committee of Award will consist of Dr. Alfred L. Loomis, Pres. N. Y. Acad. of Medicine, Chairman; Drs. H. F. Formad, Phila.; Ezra H. Wilson. Brooklyn; Geo. F. Shrady, and Jos. H. Raymond, editor BROOKLYN MED. JOURNAL.

